# Supplementary material for: Overexpression of GhKTI12 Enhances Seed Yield and Biomass Production in Nicotiana Tabacum
Source: Genes (Basel). 2022 Feb 25;13(3):426. doi: 10.3390/genes13030426 (PMC8953243; doi:10.3390/genes13030426)
Supplement: Supplementary file 1 [file genes-13-00426-s001.zip › supp/Supplementary Figure S1.pdf]

A

Compute pI/Mw

Theoretical pI/Mw (average) for the user-entered sequence:

```
        10         20         30         40         50         60
MALVVICGQP CSGKSTAAKC LAEALNESEC KQTVRIIDET SFHLDRNQSY ANMPSEKNLR

        70         80         90        100        110        120
GVLRSEVDRS VSRDNIIIVD SLNSIKGYRY ELWCLARAAG IRYCVLYCDV GESHCRKWNE

       130        140        150        160        170        180
ERREKGEAAY NDVIFEDLVR RFEKPDRRNR WDSPLFELWA HNDGVERSSI AIVDVVLYLT

       190        200        210        220        230        240
KKADSKSRDV KILQPTIATQ NTRFSEANSL YEMDRATQEV INAIVEAQSQ SIGGPLANIS

       250        260        270        280        290        300
IGQGLPNIDI SRSVGLPELR RLRRTFIKLT GQTSLSGRPP PSDSNSAKRM FVDYLNRELG

TIA
```

Theoretical pI/Mw: 8.37 / 34177.75

B

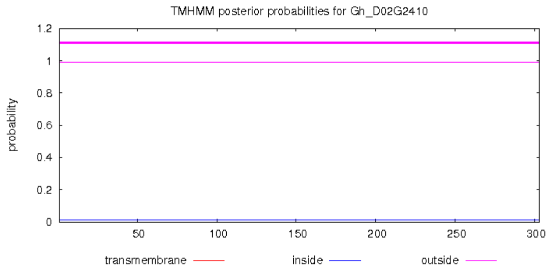

C

14 Nearest Neighbors

id	site	distance	identity	comments
PCNA_MAIZE	nucl	218.141	10.8553%	[Uniprot] SWISS-PROT45:Nuclear.
PCNA_ORYSA	nucl	230.342	12.8713%	[Uniprot] SWISS-PROT45:Nuclear.
At5g37170.1	cyto	250.3	10.479%	[Arath]
PPOL_ARATH	nucl	263.092	12.8728%	[Arath] [Uniprot] SWISS-PROT45:Nuclear. Evidence:IDA Pubmed:9778846
At3g60830.1	nucl	264.576	16.5289%	[Arath]
PCNA_CATRO	nucl	269.687	12.8713%	[Uniprot] SWISS-PROT45:Nuclear.
PCNA_DAUCA	nucl	270.985	13.8614%	[Uniprot] SWISS-PROT45:Nuclear.
At3g48040.1	plas	272.044	13.2013%	[Arath]
RPB1_ARATH	nucl	274.097	5.54348%	[Uniprot] SWISS-PROT45:Nuclear.
E13K_TOBAC	extr	277.456	13.2931%	[Uniprot] SWISS-PROT45:Extracellular.
RL11_ORYSA	cyto	279.036	14.1914%	[Uniprot] SWISS-PROT45:Cytoplasmic.
PCNA_TOBAC	nucl	283.805	11.8812%	[Uniprot] SWISS-PROT45:Nuclear.
At1g49120.1	nucl	285.345	9.42249%	[Arath]
At1g16060.1	nucl	294.234	13.6232%	[Arath]

D

Secondary Structure:

```
                   *         *         *         *         *         *
Query 1   MALVVICGQPCSGKSTAAKCLAEALNESECKQTVRIIDETSFHLDRNQSYANMPSEKNLRGVLRSEVDRS 70
Helix 1   HHHHHHHH     HHHHHHHHHHHHHHHHHHHHHHHHHHHHHH             HHHHHHHHHHHHHH 70
Sheet 1   EEEEEEEE                     EEEEEEE                                  70
Turns 1           T   TT            TT   T      TT     T T      T T       T   T  70
Struc 1   HEEEEEEETCCCTHHHHHHHHHHHHHHTHEEEEEEHHHHTHHHCCTCTCCCCCCTCTHHHHHHHHHHHTH 70

                   *         *         *         *         *         *
Query 71  VSRDNIIIVDSLNSIKGYRYELWCLARAAGIRYCVLYCDVGESHCRKWNEERREKGEAAYNDVIFEDLVR 140
Helix 71  HHHHHHHHHHH         HHHHHHHHHH               HHHHHHHHHHHHHHHHHHHHHHHHH 140
Sheet 71       EEEE        EEEEEEEE     EEEEEEEEE                    EEEEEEEEEE  140
Turns 71    TT                                     T       T     T               140
Struc 71  HHTTHEEEEHHCCCCCCEEEHEEHHHHHHHEEEEEEEEECCTCCCHHHHHHHHHHHHHHEEEEHHHEEEH 140

                   *         *         *         *         *         *
Query 141 RFEKPDRRNRWDSPLFELWAHNDGVERSSIAIVDVVLYLTKKADSKSRDVKILQPTIATQNTRFSEANSL 210
Helix 141 HHH          HHHHHHHHHHHHHHHHHHHHHHHHHHHHHHHHHHHHHHHHHHHHH             210
Sheet 141               EEEEE          EEEEEEEEEE          EEEEEEEEEEEEE         210
Turns 141    T    T   T         T   TT            T   T  T      T      T   T     210
Struc 141 HHHTCCCCTCCCTHHHHHHHHHTHHHTHHEEEEEEEEEEHHHHHTHHHHEEEEEEEEHEEEECCCTCCCC 210

                   *         *         *         *         *         *
Query 211 YEMDRATQEVINAIVEAQSQSIGGPLANISIGQGLPNIDISRSVGLPELRRLRRTFIKLTGQTSLSGRPP 280
Helix 211   HHHHHHHHHHHHHHHHHHH    HH             HHHHHHHHHHHHHHHHHHHHHHH        280
Sheet 211       EEEEEEEEE                                    EEEEEEEEEEE         280
Turns 211        T          TT   T        T        T           T      T     T    280
Struc 211 CCHHHHEEEEEHHHHHHTTHCCTCHHCCCCCTCCCCCCCHTHHHHHHHHHEEEEEEEEETEHCCCTCCC 280

                   *         *
Query 281 PSDSNSAKRMFVDYLNRELGTIA 303
Helix 281       HHHHHHHHHHHHHHHH  303
Sheet 281          EEEEE          303
Turns 281     T           T       303
Struc 281 CCCCTCHHHEEEEEHHHHHHHHC 303


Total Residues: H: 206   E: 99    T: 42
      Percent: H: 68.0  E: 32.7  T: 13.9
```

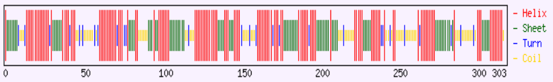

Figure S1. Bioinformatic analysis of *GhKTI12*. (A) Computation of pI/MW of *GhKTI12* by proteomics ExPASy website. (B) GhKTI12 protein TMH prediction by TMHMM 2.0 version online server. (D) Prediction of subcellular localization of *GhKTI12* analysis of the amino acid sequences (https://wolfpsort.hgc.jp) (D) Secondary Structure of GhKTI12 calculated by GOR CFSSP ExPASy online tool.
