# Supplementary material for: Overexpression of GhKTI12 Enhances Seed Yield and Biomass Production in Nicotiana Tabacum
Source: Genes (Basel). 2022 Feb 25;13(3):426. doi: 10.3390/genes13030426 (PMC8953243; doi:10.3390/genes13030426)
Supplement: Supplementary file 1 [file genes-13-00426-s001.zip › supp/Supplementary Figure S2.pdf]

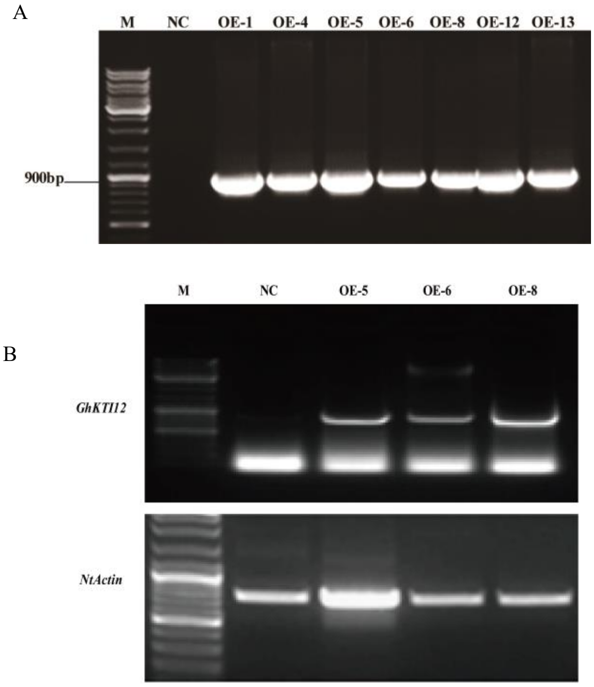

Figure S2. Identification of the *GhKTI12* transgenic plants. (A) PCR identification of seven independent T0 transgenic lines. (B) Validation of the transcript sequence encoding *GhKTI12* gene by RT-PCR analysis in OE-5, OE-6 and OE-8 transgenic lines.
