# Supplementary material for: Overexpression of GhKTI12 Enhances Seed Yield and Biomass Production in Nicotiana Tabacum
Source: Genes (Basel). 2022 Feb 25;13(3):426. doi: 10.3390/genes13030426 (PMC8953243; doi:10.3390/genes13030426)
Supplement: Supplementary file 1 [file genes-13-00426-s001.zip › supp/Supplementary Figure S3.pdf]

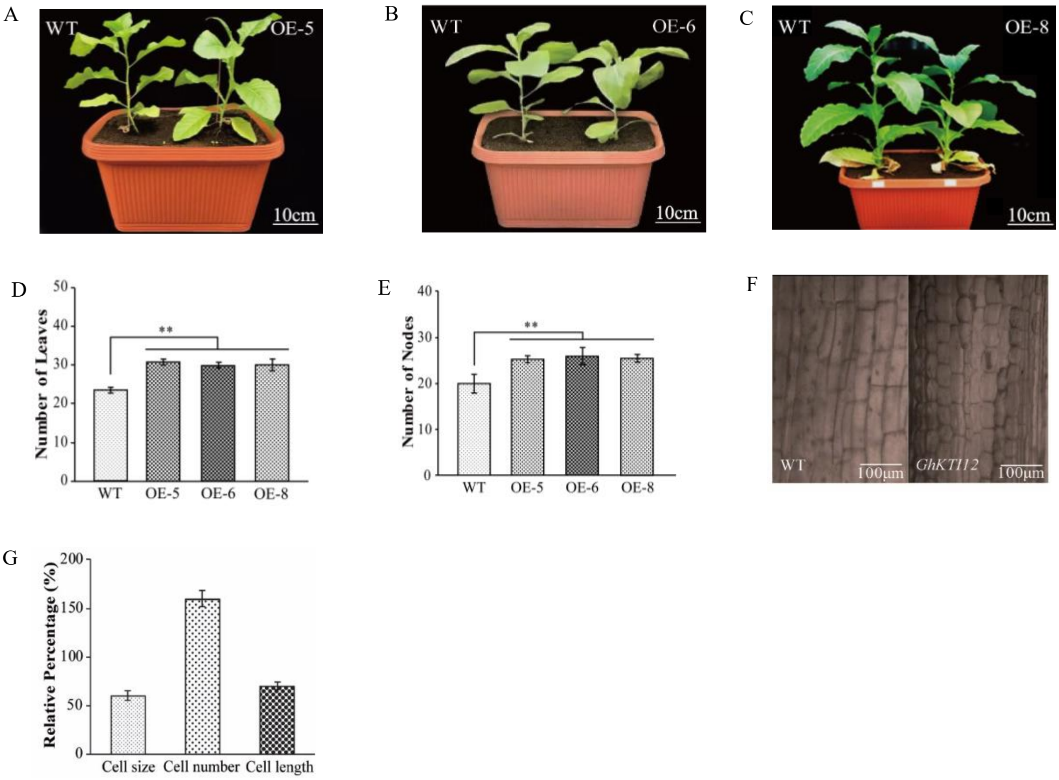

Figure S3. Morphology analysis and stem cell observation of *GhKTI12* transgenic plants. (A, B, C) Morphology comparison of WT and *GhKTI12*-tobacco before flowering time. (D)Total number of leaves per plant in the whole development stage (Scale =100 um). (E) Main stem internodes per plant in the main stem. (F) Microscopic observation of longitudinal section mainstem internode in wild type and transgenic plant. (Bars = 100 μm). (G) Relative percentage of mainstem internode parenchyma cell size, cell number and cell length in *GhKTI12* transgenic tobacco compared with wild type. Bars in the graph show the standard mean error. Asterisks indicates significant differences between wild type (WT) and *GhKTI12* transgenic plants analyzed by Student's t-test, (*) $P < 0.05$; (**) $P < 0.01$. Data were collected from 10 representative plants.
