# Supplementary material for: Overexpression of GhKTI12 Enhances Seed Yield and Biomass Production in Nicotiana Tabacum
Source: Genes (Basel). 2022 Feb 25;13(3):426. doi: 10.3390/genes13030426 (PMC8953243; doi:10.3390/genes13030426)
Supplement: Supplementary file 1 [file genes-13-00426-s001.zip › supp/Supplementary Figure S4.pdf]

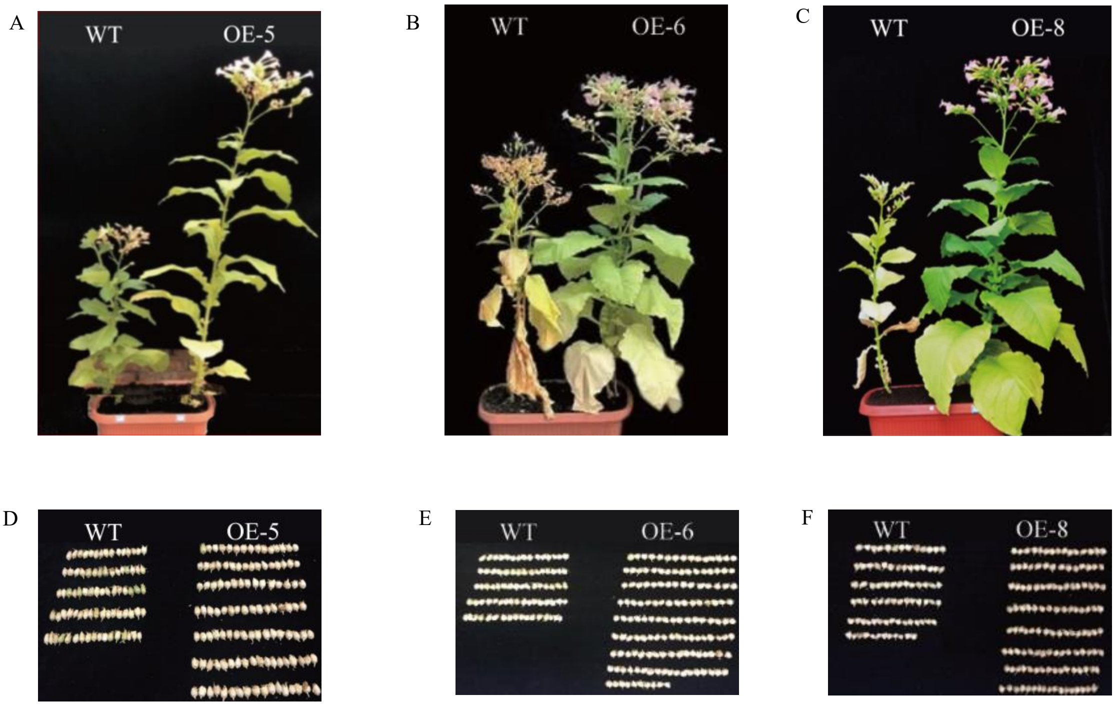

Figure S4. Observation of morphological and capsules number in *GhKTI12* transgenic plants. (A, B, C) Comparison of morphological phenotypes and (D, E, F) capsules number between wild type and *GhKTI12* transgenic plants.
