# Supplementary material for: Overexpression of GhKTI12 Enhances Seed Yield and Biomass Production in Nicotiana Tabacum
Source: Genes (Basel). 2022 Feb 25;13(3):426. doi: 10.3390/genes13030426 (PMC8953243; doi:10.3390/genes13030426)
Supplement: Supplementary file 1 [file genes-13-00426-s001.zip › supp/Supplementary Figure S5.pdf]

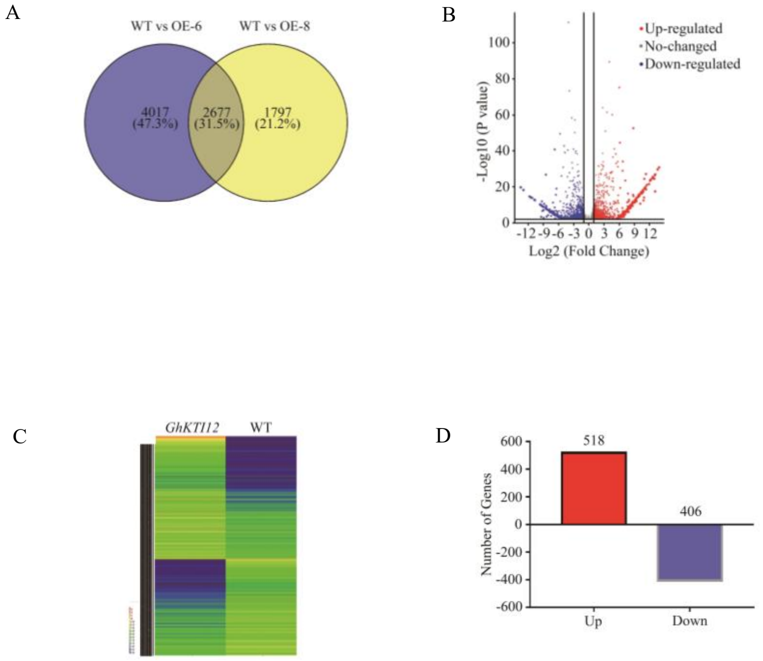

Figure S5. Analysis of differential expressed genes (DEGs) in *GhKTI12* transgenic plants. (A)Venn diagram shows the overlap of the differently expressed genes (DEGs). (B) Heatmap for cluster analysis of overlap DEGs. The color scale corresponds to the log2 (FPKM) values of genes in WT and *GhKTI12* transgenic plants. (C) Volcano plot showed differential expression pattern of up and down-regulated DEGS in OE-6, OE-8 compared with wild type (WT) control plant. (D) Number of Up and Down Regulated DEGs in *GhKTI12* transgenic plants.
