# Supplementary material for: Overexpression of GhKTI12 Enhances Seed Yield and Biomass Production in Nicotiana Tabacum
Source: Genes (Basel). 2022 Feb 25;13(3):426. doi: 10.3390/genes13030426 (PMC8953243; doi:10.3390/genes13030426)
Supplement: Supplementary file 1 [file genes-13-00426-s001.zip › supp/Supplementary Figure S7.pdf]

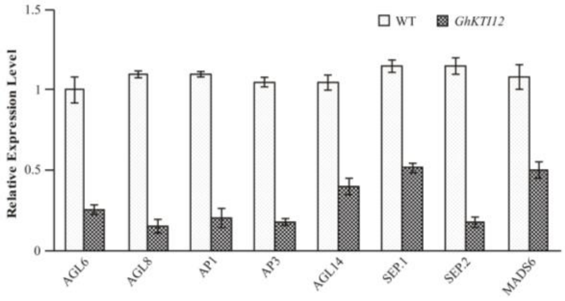

Figure S7. qRT-PCR analysis of downregulated genes related to plant flowering in *GhKTI12* transgenic plants and WT plants. Bars in the graph show the standard mean error. Asterisks indicates significant differences between wild type (WT) and *GhKTI12* transgenic plants analyzed by Student's t-test, P < 0.01.
