# Supplementary material for: Overexpression of GhKTI12 Enhances Seed Yield and Biomass Production in Nicotiana Tabacum
Source: Genes (Basel). 2022 Feb 25;13(3):426. doi: 10.3390/genes13030426 (PMC8953243; doi:10.3390/genes13030426)
Supplement: Supplementary file 1 [file genes-13-00426-s001.zip › supp/Supplementary Figures legends.pdf]

Overexpression of GhKTI12 Enhances Seed Yield and Biomass Production in *Nicotiana tabacum*

Aye Aye Myat[#1], Yu Zhou[#1], Yuan Gao[#1], Xiang Zhao[1], Chengzhen Liang[1], Muhammad Ali Abid[1], Peilin Wang[1], Umar Akram[1,2], Mubashir Abbas[1], Muhammad Askari[1], Sandui Guo[1], Rui Zhang[*1], Zhigang Meng[*1]

1 Biotechnology Research Institute, Chinese Academy of Agricultural Sciences, Beijing 100081, China

2 Institute of plant Breeding and Biotechnology, MNS- University of Agriculture, Multan Pakistan

# These authors contributed equally.

*** Correspondence: Rui Zhang, zhangrui@caas.cn; Zhigang Meng, mengzhigang@caas.cn**

Supplementary Figures

Figure legends

Figure S1. Bioinformatic analysis of *GhKTI12*.

Figure S2. Identification of the *GhKTI12* transgenic plants.

Figure S3. Morphology analysis and stem cell observation of *GhKTI12* transgenic plants.

Figure S4. Observation of morphology and capsule number in *GhKTI12* transgenic plants.

Figure S5. Analysis of differentially expressed genes (DEGs) in *GhKTI12* transgenic plants.

Figure S6. qRT-PCR analysis of cellular protein genes in *GhKTI12* transgenic plants and WT plants.

Figure S7. qRT-PCR Analysis of downregulated genes related to plant flowering in *GhKTI12* transgenic plants and WT plants.
